# Understanding the Implications of the Breast Cancer Pathology Report: A Case Study

**DOI:** 10.6004/jadpro.2013.4.3.6

**Published:** 2013-05-01

**Authors:** Susan Beikman, Patricia Gordon, Shannon Ferrari, Monica Siegel, Mary Ann Zalewski, Margaret Q. Rosenzweig

**Affiliations:** From Magee Womens Hospital, Pittsburgh, Pennsylvania

## Abstract

**Case Study**

A.K. is a 50-year-old Caucasian female who was recently diagnosed with breast cancer and is presenting for an opinion regarding adjuvant therapy following a right segmental mastectomy and sentinel lymph node biopsy. The advanced practitioner (AP) in the outpatient oncology treatment center will be expected to participate in the discussion regarding the patient’s care plan. In her review of the final pathology, the AP notes that the breast tumor is infiltrating ductal carcinoma, the most common type of invasive breast cancer. It starts in the cells that line the milk ducts in the breast, grows outside the ducts, and often spreads to the lymph nodes. Infiltrating ductal carcinoma represents 65% to 85% of all breast cancer cases (College of American Pathologists, 2011). The breast tumor measures 1.2 × 0.9 × 1.0 cm^3^ (T1), is estrogen receptor positive with an H score of 280, is progesterone receptor negative with an H score of 0, and is HER2 equivocal 2+ by immunohistochemistry with a fluorescence in situ hybridization ratio of 1.9 with a copy number of 5. The Ki-67 proliferation rate is 60%, and the nuclear grade is 2/3, with a Nottingham score of 5/9. The surgical margins from the segmental mastectomy are clear by 0.4 mm. There is lymphovascular invasion present. Of two sentinel lymph nodes, none is positive (N0). There has been no workup for metastatic disease. Additionally, the AP notes that the patient is premenopausal and that A.K.’s family history is positive for a maternal aunt diagnosed with a stage I breast cancer at age 75. What is the recommended plan of care for A.K.?

Solid tumor staging includes tumor size (T), regional lymph nodes (N), and distant metastasis (M). This TNM staging system used in solid tumor oncology is well known to oncology professionals (Edge et al., 2010). For breast cancer, stage of disease and number of positive lymph nodes influence the need for a staging workup to rule out distant metastasis. For patients with early-stage disease (I to IIB), the use of imaging studies to detect distant metastasis is not recommended unless the patient has signs or symptoms of metastasis such as bone pain, abdominal symptoms, abnormal liver function tests, or elevated alkaline phosphatase levels. For patients with locally advanced or stage IV disease, a staging workup is recommended (National Comprehensive Cancer Network [NCCN], 2013; Myers, Johnston, Pritchard, Levine, & Oliver, 2001; Puglisi et al., 2005). For example, a woman presenting with early-stage disease (I to IIB) and no positive lymph nodes would be recommended to have a chest x-ray but no further staging. For molecular evaluation, the NCCN guidelines also recommend the evaluation of estrogen (ER), progesterone (PR), and HER2 receptors for breast cancer (NCCN, 2013).

## ER/PR Status

The responsiveness of breast malignancies to endocrine therapy is an important parameter in managing the disease. The NCCN guidelines recommend that all breast cancer tumors be analyzed for ER and PR status (Hammond, Hayes, Wolff, Mangu, & Temin, 2010). The expression of these receptors identifies those patients most likely to benefit from endocrine therapy. Breast cancer cells have receptor molecules to which estrogen and progesterone will bind. These receptors can contribute to ER-positive tumor growth. Estrogen receptor–positive tumors are more likely to be histologically well differentiated (Ferrero-Poüs et al., 2001; Chu, Anderson, Fritz, Ries, & Brawley, 2001; Knoop, Bentzen, Nielsen, Rasmussen, & Rose, 2001; Wenger et al., 1993), to have a lower fraction of dividing cells, and to be diploid (Wenger et al., 1993). The estrogen positivity of a breast cancer tumor is not dependent on serum estrogen. Immunohistochemistry detects antigens in tissue by visualizing an antigen-antibody interaction.

ER/PR receptors are most commonly measured by immunohistochemistry (IHC). Quantification systems may use only the proportion of positive cells or may include the intensity of immunoreactivity. The results are reported as either an H score (a semiquantitative system for pathologists, as they assess both the intensity and distribution of positive staining) with a range between 0 and 300, a percentage between 0% and 100%, or a number 0, 1+, 2+, 3+ (Putti et al., 2005). This scoring may differ among pathology departments but regardless of the scale, the higher the number, the more receptors present on breast cancer cells.

## HER2 Status

HER2 status is obtained on all invasive breast cancer biopsies. HER2 is a member of the epidermal growth factor receptor (EGFR) family of receptors. Amplification or overexpression of its protein product is present in approximately 10% to 20% of breast cancers (Lal, Salazar, Hudis, Ladanyi, & Chen, 2004; Owens, Horten, & Da Silva, 2004; Yaziji et al., 2004). Overexpression of the HER2 protein has prognostic and treatment implications. HER2-positive breast cancer is associated with an increased rate of metastasis, a decreased time to recurrence, and a decrease in overall survival.

Accurate determination of HER2-positive disease is critical, so that appropriate therapies such as trastuzumab (Herceptin), lapatinib (Tykerb), or the newer pertuzumab (Perjeta) are utilized. Because these therapies are potentially cardiotoxic—approximately 1% to 4% of patients can develop serious cardiac toxicity from trastuzumab—it is important that these drugs be used only in patients who are definitively positive (Telli, Hunt, Carlson, & Guardino, 2007). HER2-positive cancers typically respond well to anthracycline and taxane chemotherapies but not well to cyclophosphamide chemotherapy or to tamoxifen in ER-positive disease (Villman et al., 2006; Pritchard et al., 2006; Cardoso et al., 2004; Thor et al., 1998).

In 2007, the American Society of Clinical Oncology (ASCO) and the College of American Pathologists (CAP) released guidelines for laboratory testing of HER2 on breast biopsies (Wolff et al., 2007). The goal of the guidelines was to establish maximal accuracy of HER2 testing by IHC and fluorescence in situ hybridization (FISH). There are other ways to determine HER2 positivity, such as the measurement of overexpression of HER2 RNA and reverse-transcriptase polymerase chain reaction (RT-PCR). In addition, there are proposed methods of evaluation looking at alternative reference genes on biopsies with polysomy 17. These methods are still under investigation (Tse et al., 2011). Both IHC and FISH testing will be reviewed here.

Immunohistochemistry is the most common method used to determine ER, PR, and HER2 status on breast cancer biopsies. HER2 results are subjectively graded on a scale of 0 to 3+, with 0 to 1+ consistent with low expression, 2+ equivocal, and 3+ positive for amplification. The equivocal specimens are then further evaluated by FISH analysis (Vanden Bempt et al., 2008).

Fluorescence in situ hybridization is a technique for the measurement of gene amplification that uses fluorescently labeled DNA probes. While FISH testing is more expensive, takes longer, and requires a fluorescent microscope, it is more accurate than IHC testing. FISH results are interpreted as copies of the HER2 gene per chromosome 17. If the ratio of HER2 to centromere on chromosome 17 (CEP 17) is greater than 2.2, the specimen is considered to be amplified. If the ratio is less than 1.8, the specimen is considered to be nonamplified. Ratios between 1.8 and 2.2 are considered equivocal. Equivocal HER2 testing results may be related to polysomy 17 (greater than 3 copies of the chromosome) indicated in the pathology report as the copy number. Tumors with an increased number of chromosomes 17 will contain more copies of the HER2 gene, which could elevate HER2 expression (Lester et al., 2009).

## Tumor Grade

Breast cancer tumor grade is based on both cytologic and architectural features of the breast cancer specimen (Lester et al., 2009). When determining the tumor grade of breast cancer, three areas are taken into consideration by the pathologist: tubule formation, mitotic activity, and nuclear grade. Overall, the lower the grade, the less aggressive the breast cancer. Conversely, higher grades indicate a more aggressive breast cancer.

Accoring to CAP, the Nottingham score is the current standard for grading breast cancer (Lester et al., 2009). The Nottingham score, also referred to as the Elston-Ellis modification of the Scarff-Bloom-Richardson grading system, is made up of three variables of the breast pathology (Lester et al., 2009; Elston & Ellis, 1991). Each of the three variables is scored individually on a scale of 1 to 3, with a total potential score of 3 to 9. First, the amount of tubule formation is determined and scores of 1 to 3 are determined. A score of 1 means that more than 75% of the tumor area shows glandular or tubular structures consistent with normal breast tissue. A score of 2 indicates 10% to 75% glandular or tubular structures, and a score of 3 means less than 10% of glandular/tubular structures are present.

Next, the nuclei of the cells are evaluated to determine their size and shape. Lower scores again show that the nuclei are closer to normal, and higher scores show more variability compared to normal cells. Finally, a mitotic count is determined. This looks at the number of mitotic figures found in the most active part of the tumor. These measurements are made according to a set scale, again with low mitosis counts producing lower scores and high mitosis counts producing higher scores.

These three scores are then added together for the total Nottingham score. A score of 7, 8, or 9 indicates a high-grade, more aggressive tumor; a score of 4, 5, or 6 indicates an intermediate tumor; and a score of 1, 2, or 3 indicates a less aggressive tumor (Lester et al., 2009; Elston & Ellis, 1991).

## Ki-67

The Ki-67 protein is a nuclear antigen that is expressed throughout the majority of the cell cycle. It is utilized as a measure of dividing cells, detecting cells in synthesis phase. Ki-67 is considered a cellular marker for proliferation, predicting proliferative activity of tumors. The fraction of Ki-67–positive tumor cells is often correlated with the clinical course of cancer.

Many studies have investigated the relationship between Ki-67 and prognosis in breast cancer. High Ki-67 expression is a sign of poor prognosis, but it is associated with a good chance of clinical response to chemotherapy. High Ki-67 expression has been associated with a significantly higher risk of relapse in lymph node–positive as well as lymph node–negative disease (de Azambuja et al., 2007; Dowsett et al., 2007).

In 1999, the CAP consensus statement recommended routine assessment of cellular proliferation in the evaluation of breast cancer (Fitzgibbons et al., 2000). However, since an assessment of proliferative rate is included in the Nottingham score as the mitotic score, more proliferation markers may not contribute additional information. In 2007, an ASCO expert tumor marker committee recommended against the routine use of proliferation markers to assign patients to prognostic groups. Therefore, the independent significance of the Ki-67 score is modest (Dowsett et al., 2007; Harris et al., 2007).

## Lymphovascular Invasion

Lymphovascular invasion (LVI) is defined as the invasion of lymphatic spaces, blood vessels, or both in the peritumoral area by tumor emboli. The presence of LVI is an indicator of increased risk of axillary involvement and distant metastasis. Studies have suggested that LVI by tumor cells is an adverse prognostic factor for relapse and survival in node-negative patients (Lee et al., 2006; Pinder et al., 1994; Lauria et al., 1995; Veronesi et al., 1995; Rosen, Groshen, Saigo, Kinne, & Hellman, 1989), in combination with other risk factors, including tumor grade, size, and receptor status (Soerjomataram, Louwman, Ribot, Roukema, & Coebergh, 2008). It is not clear whether the presence of LVI should be included in upstaging a patient from low to high risk. Lymphovascular invasion is not included in most internationally recognized staging systems, as it remains unclear whether its presence is an independent, high-risk criterion in clinically relevant staging subgroups. Therefore, LVI can be associated with poorer outcomes in patients already classified as having high-risk breast cancer but not in patients classified as having low-risk disease (Ejlertsen et al., 2009).

## Discussion of the Case

The plan of care for A.K. will be recommended based on ER/PR and HER2 status, lymph node status, type of surgery, and menopausal status. Baseline laboratory testing should include a complete blood count and chemistries, including liver function tests with alkaline phosphatase. Adjuvant treatment may include trastuzumab-based chemotherapy, radiation therapy, and endocrine therapies. A.K.’s chemotherapy regimen would most likely include trastuzumab because her HER2 status was equivocal by IHC and FISH analysis. Also, the copy number was elevated at 5, which points to the overexpression of the protein encoded for HER2. Because of A.K.’s segmental mastectomy, adjuvant radiation therapy would generally be required. Upon completion of radiation therapy, endocrine therapy in the form of tamoxifen would be initiated. In this ER-positive woman, tamoxifen would be used for antiestrogen therapy instead of an aromatase inhibitor because of her premenopausal status. Ovarian suppression may be considered as well if A.K.’s menstrual periods continue throughout chemotherapy (NCCN, 2013).

The AP should also know that other information seen in the pathology report may not factor specifically into the decision regarding therapy for this patient but may impact decisions in other breast cancer cases. Examples of this information are Ki-67 and LVI status. Although the high Ki-67 proliferation index and evidence of LVI do not mandate therapy, their presence suggests aggressive disease and therefore suggests the need for adjuvant chemotherapy.

A.K. would not be referred for genetic testing, as she reports no first-degree relatives with breast cancer, she is over 40 years old, and she relates no family history of ovarian or male breast cancer (U.S. Preventive Services Task Force, 2005). Following the current NCCN guidelines, A.K.’s follow-up after completing radiation therapy would include tamoxifen for 5 years, a physical exam every 3 months for 3 years, followed by exams every 6 months for 2 additional years, and annual mammograms (NCCN, 2013).

## Resources

Information regarding pathology and its implications for treatment can be confusing and overwhelming to the newly diagnosed patient and her family. Some good resources to recommend in the clinical setting for patient education are Komen for the Cure and the Abramson Cancer Center's OncoLink, though there are several websites available.

## CONCLUSION

This case study illustrates the complexity of the breast cancer pathology report. The components of the report that are important to understand for both prognostic and predictive information, beyond traditional TNM staging, are highlighted. Today’s advanced level provider needs to understand these components in order to fully participate in the care of the patient with breast cancer.
